# Understanding the structural characteristics of water-soluble phenolic compounds from four pretreatments of corn stover and their inhibitory effects on enzymatic hydrolysis and fermentation

**DOI:** 10.1186/s13068-020-01686-z

**Published:** 2020-03-11

**Authors:** Xiangxue Chen, Rui Zhai, Ying Li, Xinchuan Yuan, Zhi-Hua Liu, Mingjie Jin

**Affiliations:** 1 School of Environmental and Biological Engineering, Nanjing University of Science and Technology, 200 Xiaolingwei Street, Xuanwu District, Nanjing, 210094 China; 2 Department of Plant Pathology and Microbiology, College of Agriculture and Life Sciences, Texas A&M University, College Station, TX 77843 USA

**Keywords:** Lignocellulosic biomass, Phenolic inhibition, Pretreatment, Enzymatic hydrolysis, Fermentation

## Abstract

**Background:**

For bioethanol production from lignocellulosic biomass, phenolics derived from pretreatment have been generally considered as highly inhibitory towards enzymatic hydrolysis and fermentation. As phenolics are produced from lignin degradation during pretreatment, it is likely that the pretreatment will exert a strong impact on the structure of phenolics, resulting in varied levels of inhibition of the bioconversion process. Despite the extensive studies on pretreatment, it remains unclear how pretreatment process affects the properties of generated phenolics and how the inhibitory effect of phenolics from different pretreatment varies on enzymatic hydrolysis and fermentation.

**Results:**

In this study, the structural properties of phenolic compounds derived from four typical pretreatment [dilute acid (DA), liquid hot water pretreatment (LHW), ammonia fiber expansion (AFEX) and alkaline pretreatment (AL)] were characterized, and their effect on both enzymatic hydrolysis and fermentation were evaluated. The inhibitory effect of phenolics on enzymatic hydrolysis followed the order: AFEX > LHW > DA > AL, while the inhibitory effect of phenolics on *Zymomonas mobilis* 8b strain fermentation followed the order: AL > LHW > DA > AFEX. Interestingly, this study revealed that phenolics derived from AFEX showed more severe inhibitory effect on enzymatic hydrolysis than those from the other pretreatments at the same phenolics concentrations (note: AFEX produced much less amount of phenolics compared to AL and DA), while they exhibited the lowest inhibitory effect on fermentation. The composition of phenolics from different pretreatments was analyzed and model phenolics were applied to explore the reason for this difference. The results suggested that the amide group in phenolics might account for this difference.

**Conclusions:**

Pretreatment process greatly affects the properties of generated phenolics and the inhibitory effects of phenolics on enzymatic hydrolysis and fermentation. This study provides new insight for further pretreatment modification and hydrolysate detoxification to minimize phenolics-caused inhibition and enhance the efficiency of enzymatic hydrolysis and fermentation.

## Background

Bioconversion of lignocellulosic biomass to bioethanol has been considered as a promising strategy to circumvent global warming and solve the issue of long-term global energy insecurity [1]. Due to the recalcitrance of biomass, pretreatment is needed to disrupt the cell wall structure and increase the accessibility of cellulose to cellulases [2]. Although pretreatment is necessary for efficient enzymatic hydrolysis and ethanol production, it releases various soluble degradation products that may significantly inhibit hydrolytic enzymes and fermenting microorganisms [3, 4]. The pretreatment-derived degradation products include phenolics (lignin derivatives), furfural, acetic acid, hydroxymethyl furfural (HMF), etc. The effect of furfural, acetic acid and hydroxymethyl furfural (HMF) on enzymatic hydrolysis and fermentation has been widely studied. For example, it has been reported that acetic acid showed no significant inhibition on cellulases at a comparatively low concentration of 2 g/L, while furfural inhibited cellulases at the same concentration. In addition, it has been found that 1.4 g/L of the furans significantly delayed both glucose and xylose fermentation and HMF at same concentration showed a stronger inhibitory effect on glucose fermentation than furfural did [5]. In addition, the inhibitory effect of xylo-oligosaccharides derived from pretreatment on enzymatic hydrolysis of cellulose has been widely studied [6–8]. It has been found that cellulase inhibition by xylo-oligosaccharides was dependent on concentrations and structures. At relatively high concentrations (2–6 g/L), xylo-oligosaccharides reduced the hydrolytic activities of key cellulases and significantly inhibited cellulose hydrolysis [8, 9]. Different from these degradation products, the water-soluble phenolic compounds mainly derived from lignin have been considered as highly inhibitory compounds, which could strongly inhibit both enzymatic hydrolysis and fermentation at very low concentrations [10, 12]. However, the studies on phenolics inhibition studies were quite limited; it remains unclear that how the inhibitory effect of phenolics varies dependent on pretreatments and how they may affect both enzymatic hydrolysis and fermentation.

In order to develop more efficient pretreatment or detoxification methods for improving the bioconversion performance, a better understanding of the effect of pretreatment-derived phenolics on enzymatic hydrolysis and fermentation is desirable.

Acid pretreatment-derived phenolics have strong inhibitory effects on cellulose hydrolysis and fermentation. During dilute acid (DA) pretreatment, lignin is subjected to depolymerization and repolymerization reactions, giving rise to various types of water-soluble phenolic compounds, such as vanillin, ferulic acid and *p*-coumaric acid [11, 12]. Previous studies have shown that vanillin at a concentration of 10 mg/mL strongly decreased the cellulose hydrolysis by 26% compared to the control [13]. In addition, phenolics were also regarded as more toxic compounds towards fermenting microorganisms compared to other degradation products such as furan aldehydes and weak acids. Ezeji et al. reported that ferulic acid and *p*-coumaric acid at a concentration of 1 g/L strongly inhibited the cell growth of *Clostridium beijerinckii* BA101 bacteria strain by up to 74% [14]. Adeboye et al. found that ferulic acid (1.8 mM) and *p*-coumaric acid (9.7 mM) decreased cell growth of a *Saccharomyces cerevisiae* by up to 80% [15]. The strong toxicity of phenolics towards microorganisms might be due to their aromatic properties that make them more easily penetrate cell membranes, resulting in the damage of internal structures, the decrease in cell growth and the change of cell morphology [16].

Even though some studies have reported the effects of dilute acid (DA) pretreatment-derived phenolics, only a few studies focused on the inhibitory effect of phenolics derived from other pretreatments such as alkaline pretreatment (AL), ammonia fiber expansion (AFEX) and liquid hot water pretreatment (LHW) on enzymatic hydrolysis and fermentation. Although these pretreatments (AL, AFEX and LHW) effectively deconstruct lignocellulosic biomass, they applied different reaction mechanisms to generate phenolics from partial lignin degradation. For example, alkaline pretreatment using NaOH as the pretreatment agent results in the breakage of β-O-4 linkage, α-ether linkage and demethylation via nucleophilic substitution [17], and thus it will give rise to a high proportion of phenolics with methyl group removed. During AFEX, ammonolytic cleavage of cell wall ester and ether linkages lead to the formation of acetamide and various phenolic amides [18]. For LHW, an elevated level of hydronium ions acted as an acid at high holding temperature, to promote the partial lignin depolymerization [19]. The degree of depolymerization of lignin by LHW was much lower than that from AL pretreatment. DA pretreatment catalyzes the degradation of lignin through acid catalysis mechanism, while a high level of lignin condensation tends to happen during the pretreatment process [12]. Because of the different reaction mechanisms of these pretreatments, it is expected that different pretreatment produces phenolics with various structures, which may show varied inhibitory effects on cellulose hydrolysis and fermentation. Moreover, the pH of AL, AFEX, LHW and DA ranges from high to low. Due to the pH variety during pretreatment, the potential lignin degradation reactions might be different, which will give rise to phenolics with different effects on enzymatic hydrolysis and fermentation. For example, Humpula et al. have reported the phenolics-rich hydrolysate from AFEX-pretreated corn stover inhibited enzymatic hydrolysis by around 20% [18]. In terms of fermentation, Kim et al. found that the effect of phenolics derived from mild alkaline treatment with aqueous ammonia solution was less inhibitory [20]. However, the effect of phenolics derived from different pretreatments on fermentation has not been well evaluated and compared, as most of the previous studies used pretreatment hydrolysate, which contained various soluble compounds, or phenolic model compounds that cannot represent the actual pretreatment-derived phenolics. To further understand the role of phenolics in impeding the production of cellulosic ethanol through hydrolysis and fermentation, more detailed studies are still in need to understand the phenolics-caused inhibition. Most importantly, it is necessary to know how pretreatment may affect the characteristics of phenolics and their inhibition on the overall bioconversion processes to produce cellulosic ethanol. This knowledge will be essentially helpful for the development of less “toxic” pretreatment or detoxification process for both hydrolysis and fermentation and the improvement of ethanol bioconversion performance.

The inhibitory effects of water-soluble phenolic compounds derived from AL, DA LHW and AFEX-pretreated corn stover on both enzymatic hydrolysis and sugar fermentation by *Zymomonas mobilis* 8b strain were systematically evaluated in this work. The structure characteristics of the phenolics were conducted by using LC–MS and FT-IR to elucidate the differences in the phenolics derived from different pretreatments. The effects of typical model phenolics compounds from pretreatment process on enzymatic hydrolysis and fermentation were also studied.

## Results and discussion

### Mass balance of water-soluble phenolic compounds (WPC)

**Fig. 1:**
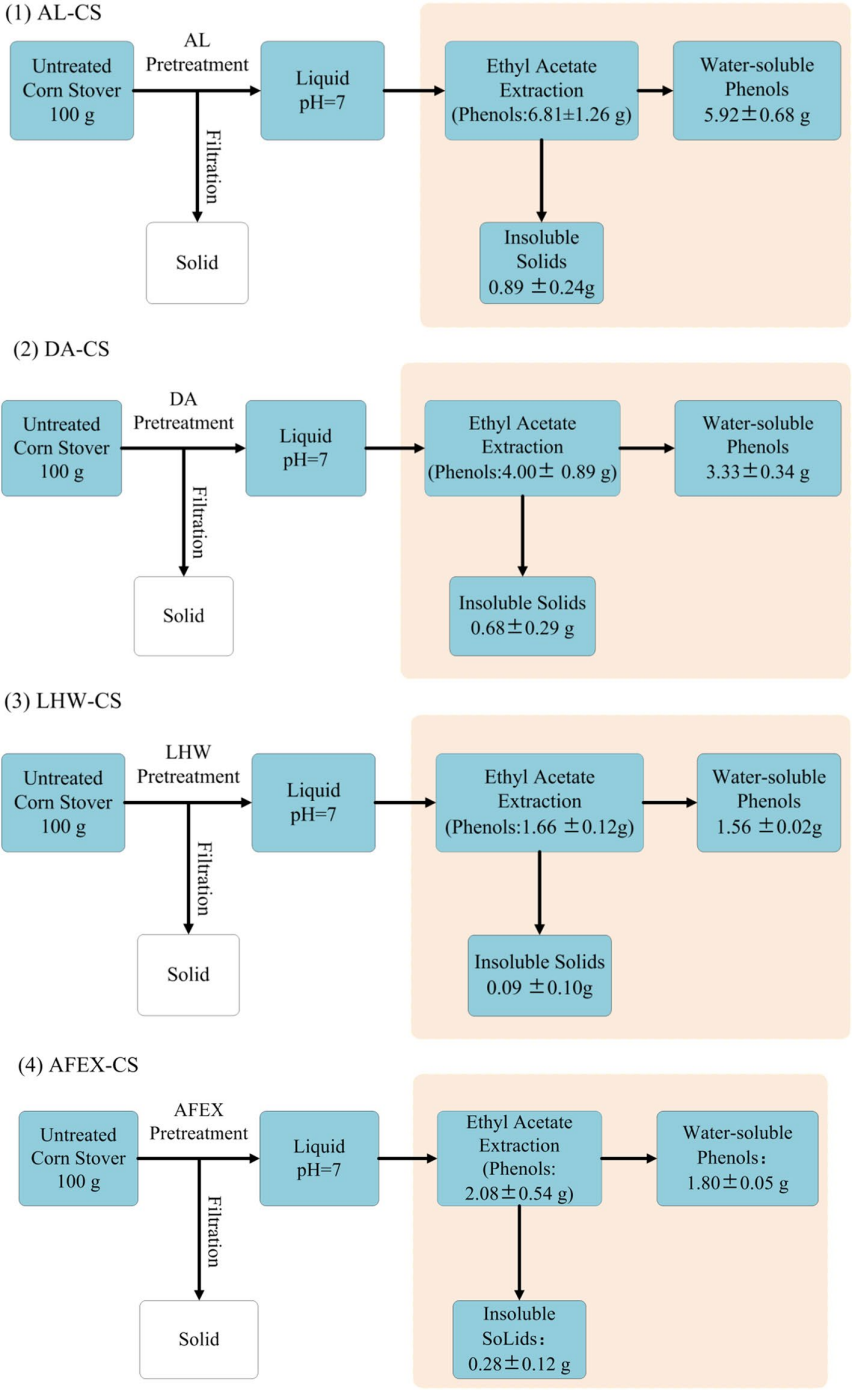
Ethyl acetate extraction of phenolics from different pretreated corn stover. (1) AL-CS: alkali pretreated corn stover; (2) DA-CS: dilute acid pretreated corn stover; (3) LHW-CS: liquid hot water pretreated corn stover and (4) AFEX-CS: ammonia fiber expansion pretreated corn stover. Phenolics were determined according to Folin Ciocalteu assay, and vanillin was used as standard for this method to measure the phenolics content. The phenolics measurement was done in duplicate with the average values and standard deviations reported

During pretreatment, various phenolics were generated from lignin and reported to be inhibitory toward enzymatic hydrolysis and fermentation [19, 21, 22]. In addition, the composition and structure of phenolics also significantly depended on the pretreatment conditions [23, 24]. In the present work, different pretreatments were conducted under optimum conditions, and then phenolics were extracted from pretreatment liquid stream through solvent extraction. Four pretreatments, including DA, LHW, AFEX and AL were selected to represent pretreatments at various pH. Results showed that pretreatments had a great impact on the amount of WPC generated (Fig. 1). According to the composition analysis, it was found that more than 80% of the extractives from pretreatment liquid streams were WPC (Fig. 1). Specifically, after pretreatment of 100 g corn stover, AL produced 5.9 g WPC, while DA released 3.3 g WPC. LHW and AFEX produced 1.6 g and 1.8 g WPC, respectively, which were much less compared with AL and DA. Although the WPC contained a minor amount of sugars and other degradation compounds (Additional file 1: Table S1), these compounds were present at low concentrations that would not affect enzymatic hydrolysis and fermentation [9, 12, 25].

### The inhibitory effect of WPC on enzymatic hydrolysis

**Fig. 2:**
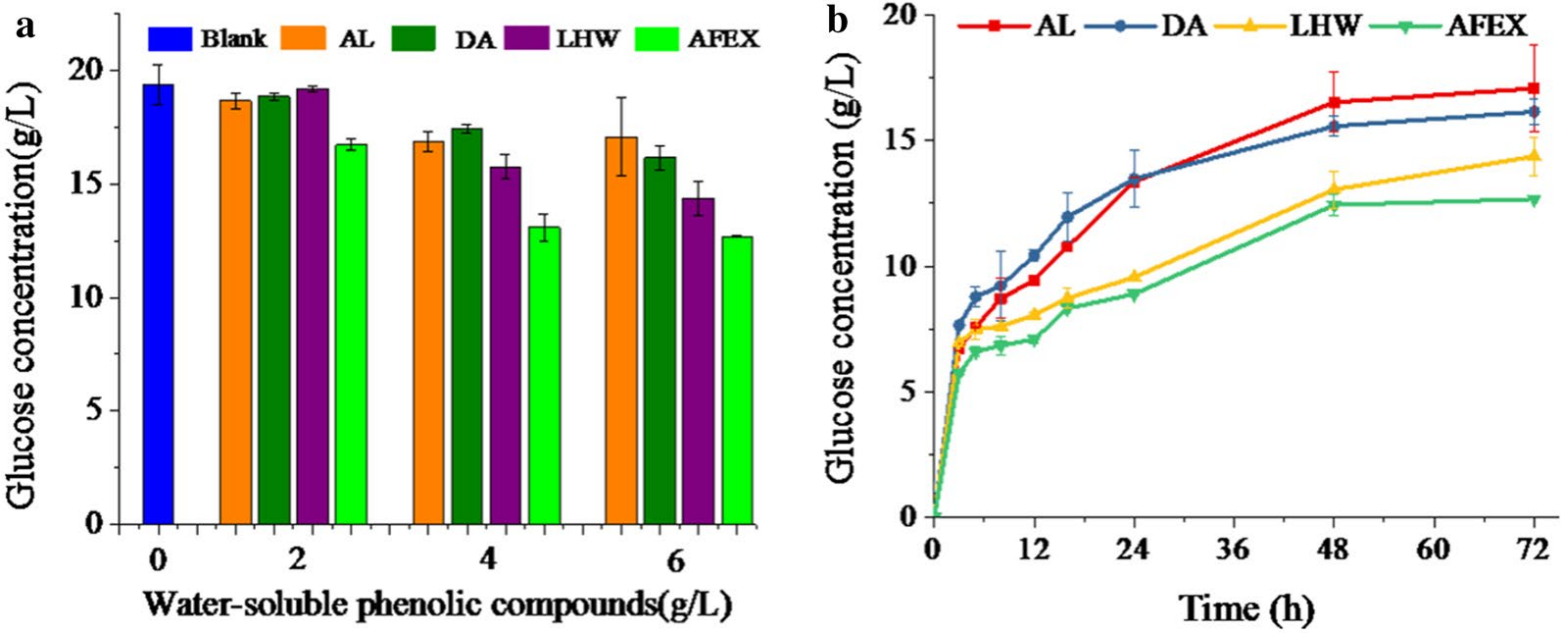
Effect of water-soluble phenolic compounds (WPC) on enzymatic hydrolysis. **a** Enzymatic hydrolysis in the presence of 0 g/L, 2 g/L, 4 g/L and 6 g/L of WPC for 72 h, at 50 °C, 25 rpm; **b** time course of enzymatic hydrolysis with 2 g/L WPC derived from each pretreatment

The effect of WPC from each pretreatment on enzymatic efficiency of cellulose was evaluated under different concentrations (Fig. 2). With the increase of the WPC concentration, the glucose yield decreased significantly in enzymatic hydrolysis. When the concentration of WPC reached 6 g/L, the efficiencies of cellulose hydrolysis were decreased by 20%–40% as compared with the control, indicating that the WPC concentration had a great impact on the inhibitory effect. In addition, the degree of inhibition by WPC highly depended on pretreatments employed. Results showed that WPC at 2 g/L derived from AFEX reduced the glucose yield by 13.8%, while WPC at the same concentration derived from LHW, AL and DA showed no significant effect on enzymatic hydrolysis. When the WPC concentration increased to 6 g/L, WPC derived from AFEX decreased the glucose concentration by 34.6%, while WPC, derived from AL, DA and LHW reduced the glucose yield by 11.9%, 16.7% and 25.8%, respectively. Interestingly, results indicated that the AFEX-WPC showed the strongest inhibitory on cellulose hydrolysis, followed by LHW-WPC, DA-WPC and AL-WPC.

To further confirm the observation in a realistic scenario, the influence of phenolics on enzymatic hydrolysis of alkaline pretreated corn stover (washed and containing both cellulose and hemicellulose) was evaluated at relatively high solid loading (20% w/w). In terms of the effect of phenolics on cellulose conversion, a similar trend was observed: AFEX-WPC showed the strongest inhibitory effect, followed by LHW-WPC, DA-WPC and AL-WPC (Additional file 1: Fig. S1). At 20% solid loading of pretreated corn stover, AFEX-WPC at 6 g/L reduced the glucose concentration from 105.1 g/L to 79.9 g/L (corresponding to 24.0% inhibition of enzymatic hydrolysis). Different from cellulase inhibition, phenolics derived from different pretreatment had similar inhibitory effects on xylose conversion (Additional file 1: Fig. S1). It could be possible that the inhibition mechanism of cellulase is different from that of xylanases or the inhibitory effect of WPC on xylanases was masked by strong sugar inhibition at high solid loading.

Previous studies have confirmed that DA and LHW derived phenolics had strong inhibitory compounds for cellulase enzymes [11, 19], however, few studies had reported the inhibitory effect of WPC derived by AFEX on cellulases. In the present study, although WPC derived from AFEX showed a stronger inhibitory effect on cellulases compared to other pretreatments, the total amount of phenolics released by AFEX was significantly lower than that generated from DA and AL. As shown in Fig. 1, AFEX generated 1.80 g WPC from 100 g corn stover, while AL and DA pretreatment released 5.92 g and 3.33 g WPC, respectively. With the consideration of the amount of WPC generated from each pretreatment, the overall inhibitory effect of WPC derived from AFEX towards enzymatic hydrolysis of cellulose was similar to that of DA-WPC and AL-WPC, while WPC derived from LHW showed the least inhibitory effect (Fig. 2). Therefore, these results showed that the inhibitory effects of WPC on the enzymatic hydrolysis depended on the concentration and pretreatment employed, and WPC derived from LHW exhibited less inhibitory effects as compared with other pretreatments.

### The inhibitory effect of WPC on fermentation

**Fig. 3:**
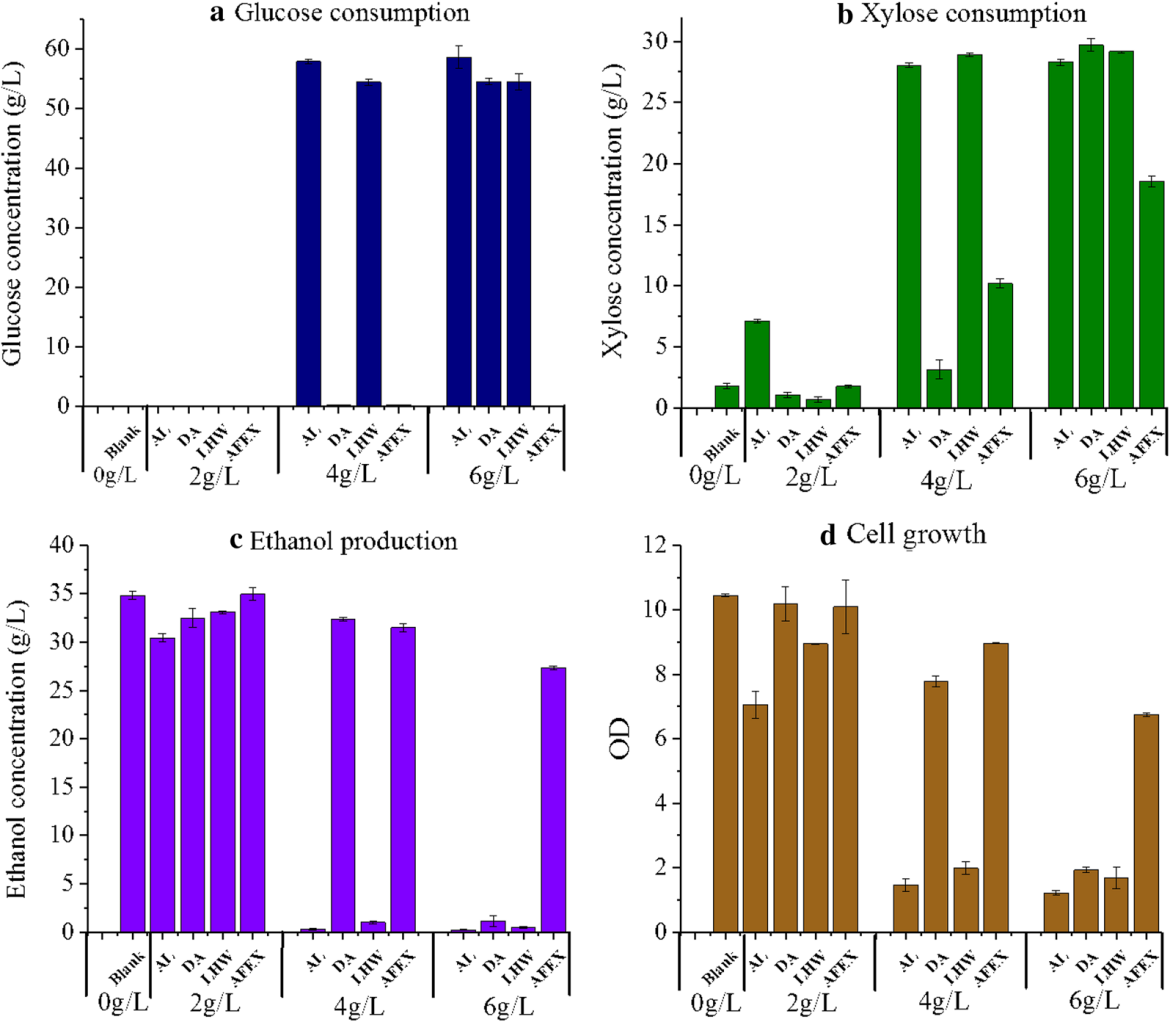
Fermentation performance of *Z. mobilis* 8b strain in the presence of 0 g/L, 2 g/L, 4 g/L and 6 g/L WPC derived from different pretreatments. **a** Residual glucose after 120 h fermentation; **b** residual xylose after 120 h fermentation; **c** ethanol titer after 120 h fermentation and; **d** cell growth. Fermentation was conducted in Erlenmeyer flasks (8 mL) at pH 6, 30 °C and 150 rpm

The effect of WPC produced from each pretreatment on ethanol fermentation was also evaluated in the present study. *Z. mobilis* 8b had been genetically modified to co-ferment glucose and xylose for ethanol production, and is featured with excellent industrial characteristics, such as low cell biomass formation, high ethanol yield and wide pH tolerance [26, 27]. Therefore, it was selected to evaluate the inhibitory effect of WPC on fermentation. As shown in Fig. 3, the inhibitory effects of WPC were also dependent on the pretreatment employed. AL-WPC showed the strongest inhibitory effect on fermentation, followed by LHW-WPC, DA-WPC, and AFEX-WPC. The inhibitory effects of WPC were greatly different from those on enzymatic hydrolysis. With the presence of 4 g/L WPC, sugar consumptions, cell growth and ethanol production were strongly inhibited by WPC derived from AL and LHW, while they were slightly inhibited by WPC derived from DA and AFEX. When the concentration increased to 6 g/L, DA-WPC exhibited almost complete inhibition of fermentation. Interestingly, AFEX-WPC at 6 g/L showed relatively low inhibitory effect on fermentation. All of the glucose and 38% of initial xylose were consumed after 120 h fermentation in the presence of AFEX-WPC, while less than 10% of initial glucose was consumed in the presence of 6 g/L WPC from other pretreatments. Growth and sugar fermentation of *Z. mobilis* 8b were most strongly inhibited by AL-WPC (Fig. 3). The difference in the WPC inhibition on fermentation might be related to molecular size and structures of phenolics generated from each pretreatment [28].

**Fig. 4:**
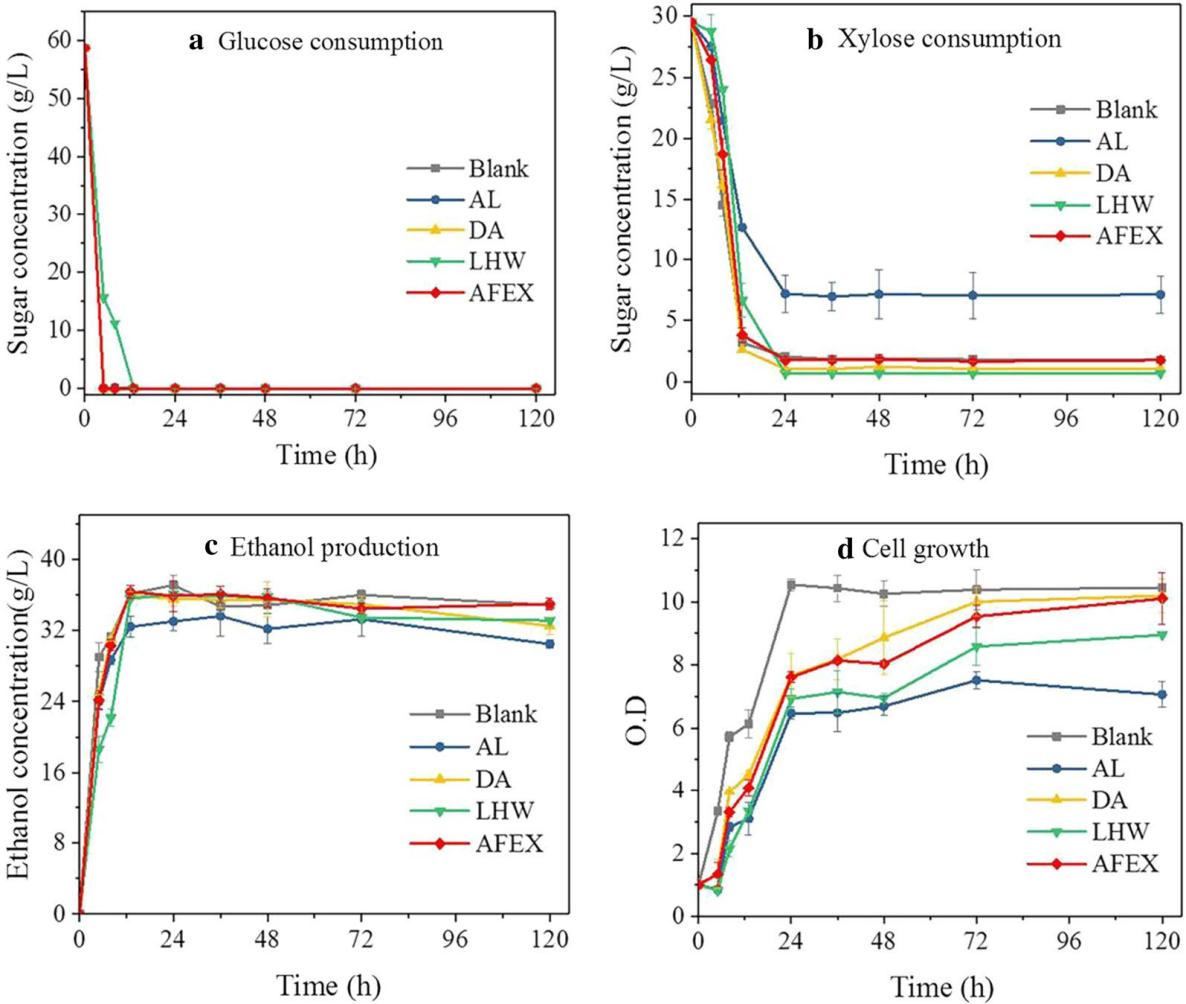
Fermentation performance of *Z. mobilis* 8b strain in the presence of 2 g/L WPC derived from different pretreatment. **a** Glucose consumption; **b** xylose consumption; **c** ethanol production and **d** cell growth. Blank: no water-soluble phenol. *AL* alkali pretreatment, *DA* dilute acid pretreatment, *LHW* liquid hot water pretreatment, *AFEX* ammonia fiber expansion (AFEX) pretreatment. Fermentation was conducted in Erlenmeyer flasks (8 mL) at pH 4.8, 30 °C and 150 rpm with inoculum at 1 (OD_600_)

WPC from each pretreatment also showed a stronger inhibitory effect on xylose consumption compared to glucose consumption (Fig. 3). Such effect was dependent on the concentration of WPC and became more obvious with the increase of WPC concentration. The fermentation time course in the presence of 2 g/L WPC is shown in Fig. 4. Glucose/xylose consumption and ethanol production were rapid in the first 24 h. At 2 g/L, AL-WPC already rendered significant reduction on xylose consumption and cell growth (Fig. 4b, d) with 7.5 g/L xylose left unfermented. In addition, the xylose consumption was sensitive to WPC, which showed a similar trend to the cell growth. Specifically, AL-derived WPC showed an obvious inhibitory effect on both the xylose consumption and cell growth at 2 g/L, while no obvious effect on glucose consumption was observed. These results suggested that the phenolics exhibited a stronger inhibitory effect on the growth of *Z. mobilis* 8b strain and xylose consumption. Considering the significant inhibitory effect of phenolics on xylose consumption, it is desirable to promote strain improvement on xylose utilization, especially in the presence of pretreatment-derived phenolics.

### Characteristics of WPC

The above-mentioned results suggested that WPC derived from different pretreatments had diverse inhibitory effects on enzymatic hydrolysis and fermentation. To investigate the potential inhibitory mechanism of WPC, the composition and structure characteristics of WPC derived from each pretreatment were evaluated. According to LC–MS analysis (Additional file 1: Table S2), AL-WPC and AFEX-WPC contained a high percentage of *p*-coumaric acid and benzaldehyde. According to previous studies, *p*-coumaric acid was esterified and linked to hemicelluloses or lignin in grasses and it could be released through alkaline pretreatment; in addition, benzaldehyde was found to be the major product generated from breakage of the β-O-4 model compound via nucleophilic substitution under basic hydrothermal conditions [29, 30]. Different from AL-CS, feruloylamide was detected in the WPC from AFEX-CS, which is consistent with previous observation [31]. Feruloylamide has been considered as a major ammoniated lignin degradation product produced by ammonolysis of polyphenolic esters. For the WPC from LHW-CS, the percentage of *p*-coumaric acid reduced and the percentage of benzoic acid increased compared to AL and AFEX, suggesting the degradation of esterified *p*-coumaric acid was limited under hydrothermal conditions. For the WPC from DA-CS, *p*-coumaric acid was the predominant product, with a relatively high amount of benzaldehyde. According to previous studies, benzaldehyde was suggested to be the product derived from the acidolysis of lignin reported by previous studies [11].

**Fig. 5:**
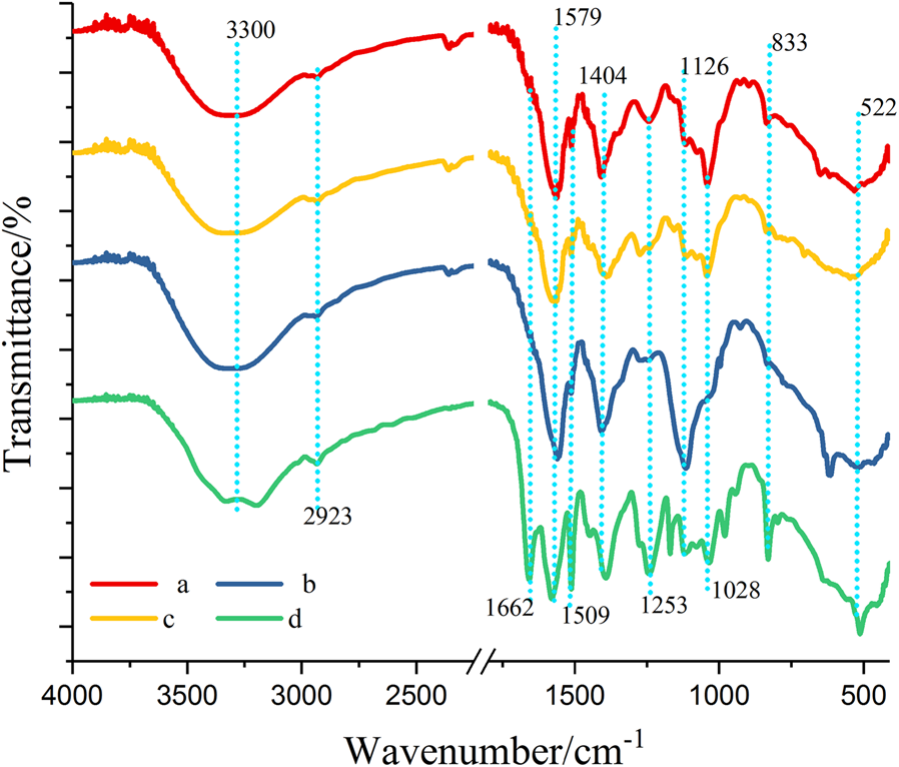
FT-IR spectra of WPC derived from different pretreatments. **a** AL; **b** DA; **c** LHW; **d** AFEX. *AL* alkali, *DA* dilute acid, *LHW* liquid hot water, *AFEX* ammonia fiber expansion

To comprehensively understand the characteristics of WPC, FT-IR was employed for functional group analysis of phenolics to provide a general outlook of the major structure properties of WPC (Table 1 and Fig. 5). Results showed that the four types of WPC exhibited similar adsorption spectrum at 3300, 2923, 1509 cm^−1^, which could be assigned to the stretching vibrations of aromatic hydroxyl groups, C–H stretching vibrations from methyl and methylene residue and stretch of aromatic rings, respectively [32]. As the strong peak at 1509 cm^−1^ shared by those four WPC suggested the presence of aromatic rings, this peak could be used as the reference peak to semi-quantitatively and analyze the relative amount of different functional groups [33, 34].

**Table 1:** FT-IR analysis of WPC derived from AL, DA, LHW and AFEX pretreatment

Wavenumber/cm^−1^	Origin	Attribution and description of FT-IR absorption	Wavenumber cm^−1^			
			AL-WPC	DA-WPC	LHW-WPC	AFEX-WPC
3200–3400	O–H	Wide absorption band, aliphatic and aromatic O–H stretching	3308	3308	3308	3308
3350–3200	CONH_2_	[36], N–H absorption, double peak	NA	NA	NA	3210 and 3348
2908–2992	CH_3_, CH_2_	Aliphatic and aromatic O–H stretching	2925	2925	2925	2939
1650–1660	CONH, CONH_2_	C=O stretching vibration	NA	NA	NA	1654
1610–1550 and 1420–1300	COO–	Carboxylate	1567 (1410)	1567 (1410)	1567 (1410)	1567 (1410)
1600 (1450)	Ar	Aromatic hydrocarbon	1600 (1450)	1600 (1450)	1600 (1450)	1600 (1450)
1507–1515		Aromatic skeletal vibration; G > S	1509	1509	1509	1509
1267	O–, CO–O	G ring and acyl-oxygen bond CO–O stretching vibration	NA	NA	1267	NA
1237–1241	OCH_3_	Methoxyl, C–C and C–O stretching vibration; C]O stretching vibration (condensed G > etherified G)	1235	NA	1239	1236
1168–1170	COOC	HGS characteristics; conjugated ester-based C=O stretching vibration	NA	NA	NA	1169
1126–1127	C–H, C–O	C–H aromatic-plane bending vibration, characteristics of S ring; coincide with secondary alcohol C–O stretching vibration	1127	1126	1126	1127
1035–1045	C–H, C–O	C–H aromatic in-plane bending vibration, G > S; primary alcohol C–O stretching vibration	1042	NA	1042	1038
834–835	C–H	S ring C–H out-of-plane bending vibration	835	NA	NA	835

In the spectrum of WPC derived from AFEX (Table 1 and Fig. 5), a high-intensity peak at 1662 cm^−1^ displayed, which could be attributed to the presence of CONH_2_ in amide groups [36]. This is consistent with the LC–MS result. In addition, previous study had confirmed that the phenolics in AFEX-pretreated biomass contained a large amount of amides, such as acetamide, ferulamide and coumaroylamide due to ammonolysis of lignin by using ultra-high performance liquid chromatography–mass spectrometry (UHPLC–MS) [31]. The shoulder peak at 1662 cm^−1^ in the spectrum of DA-WPC, LHW-WPC and AL-WPC suggests the amount of free –C=O or CONH2 [34, 35]. Interestingly, as shown in Table 2, the ratio of Abs1662 to Abs1509 followed the order: R_AFEX_ > R_LHW_ > R_DA_ > R_AL_, which was consistent with the degree of inhibition of cellulose hydrolysis by the relevant WPC. It had been reported that the –C=O or CONH_2_ served as strong dipole and could strongly interact with cellulases to inhibit cellulase activity [36, 37]. Thus, the higher amount of –C=O or CONH_2_ might result in stronger binding between phenolics and enzymes and reduction of enzymatic hydrolysis performance. Previous studies showed the contribution of –C=O to phenolic inhibition on cellulase, however, whether CONH_2_ is responsible for the cellulase inhibition remains unknown. Based on the correlation observed above, it is likely that the CONH2 group in WPC generated from AFEX also contributed to the cellulase inhibition.

**Table 2:** Relevant FT-IR absorption bands and related absorbance ratios (other’s absorbance/1509 cm^−1^’s absorbance) of WPC

Band (cm^−1^)	AL-WPC	DA-WPC	LHW-WPC	AFEX-WPC
Baseline corrected band intensities				
3300	0.69	0.81	0.80	0.45
1662	0.38	0.44	0.60	1.00
1579	1.77	1.69	2.00	1.21
1509	1.00	1.00	1.00	1.00
1404	1.54	1.56	1.50	1.00
1253	0.77	0.75	1.00	0.90
1168	0.54	1.19	0.70	0.79
1126	1.00	2.00	1.00	0.76
1028	1.46	1.13	1.30	0.86
833	0.85	0.75	0.80	0.83

In terms of the effect of WPC derived from each pretreatment on fermentation, AL-WPC showed the strongest inhibitory effect likely due to the high amount of phenolic aldehyde, which had been confirmed as inhibitors of fermenting microorganisms [38]. Considering the structural characteristics of phenolics, it was reported that the amide group in phenolics lowered the toxicity of phenolics towards *Saccharomyces cerevisiae* 424A (LNH-ST) [39]. Therefore, the phenolic amides generated from AFEX pretreatment might have contributed to the reduced inhibition of fermentation by *Z. mobilis* 8b, as compared with other WPC generated from AL, DA, and LHW pretreatment.

### Effect of phenolics with acid and amide group on enzymatic hydrolysis and fermentation

**Fig. 6:**
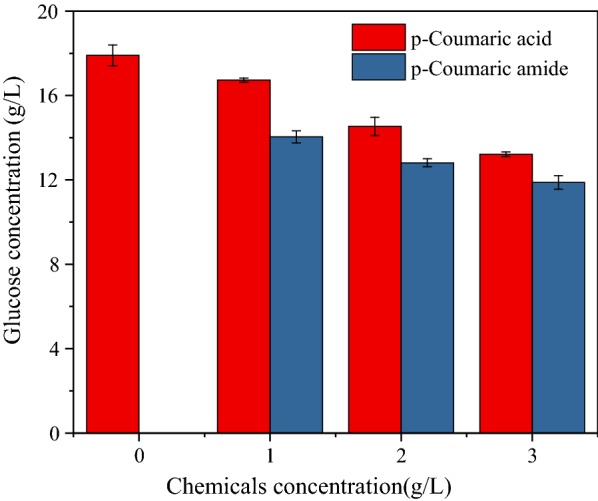
Effect of *p*-coumaric acid and *p*-coumaric amide on enzymatic hydrolysis of Avicel. Enzymatic hydrolysis with different concentrations of *p*-coumaric acid and *p*-coumaric amide was performed for 72 h, at 50 °C, 25 rpm

The above results showed that AFEX-derived phenolics exhibited a stronger inhibitory effect on enzymatic hydrolysis but less inhibitory effect on fermentation. Based on the structure analysis, it was hypothesized that phenolic amide might contribute to such observation. To further verify the hypothesis, model phenolics were used as inhibitors for enzymatic hydrolysis and fermentation. *p*-Courmaric amide and its corresponding acid were selected as typical phenolic compounds. Figure 6 shows that the glucose release in the presence of *p*-coumaric acid and *p*-coumaric amide decreased gradually with the increase of phenolic concentration. Glucose release was significantly inhibited by *p*-coumaric amide, as glucose concentration was reduced by 21.6%–33.6% in the presence of 1.0–3.0 g/L *p*-coumaric amide as compared with the reduction of 6.6%-26.2% in the presence of 1.0–3.0 g/L *p*-coumaric acid. These results suggested that phenolics with amide groups might have a stronger inhibitory effect compared with phenolics with acidic groups.

**Fig. 7:**
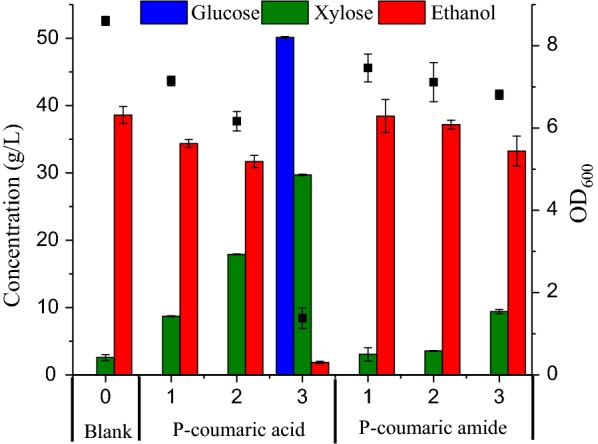
Fermentation performance of *Z. mobilis* 8b strain in the presence of 0 g/L, 1 g/L, 2 g/L and 3 g/L *p*-coumaric acid or *p*-coumaric amide. Fermentation was conducted in Erlenmeyer flasks at pH 6, 30 °C and 150 rpm for 120 h. Medium containing 60 g/L glucose, 30 g/L xylose, 5 g/L yeast extract, 10 g/L tryptone was used for fermentation

To further study why AFEX-derived phenolics showed low inhibitory effect on fermentation by *Z. mobilis* 8b, the effects of model phenolics (*p*-coumaric acid and *p*-coumaric amide) on fermentation were studied. As expected, *p*-coumaric amide had a lower inhibitory effect on fermentation as compared with *p*-coumaric acid (Fig. 7). With increase of phenolics concentration, *p*-coumaric acid at 3 g/L significantly decreased the ethanol concentration by 95.2%, while *p*-coumaric amide at 3 g/L only slightly reduced the ethanol concentration by 13.9%. The *p*-coumaric acid at 3 g/L significantly decreased both glucose and xylose consumption by *Z. mobilis* 8b strain, while *p*-coumaric amide only slightly reduced the xylose consumption (Fig. 7). The reduced sugar consumption was likely associated with the low cell growth rate, which is inhibited by the phenolics. For example, after 120 h fermentation, the OD_600_ only slightly went up to 2 in the presence of 3 g/L *p*-coumaric acid, while it reached around 7 in the presence of 3 g/L *p*-coumaric amide (Fig. 7). The result suggested the amide group of phenolics might have reduced inhibitory effect on fermentation. Therefore, these results indicated that the presence of high percentages of phenolics with amide groups in WPC derived from AFEX might contribute to their lower inhibitory effects on fermentation by *Z. mobilis* 8b strain, compared with other WPC.

## Conclusions

Results showed that the inhibitory effect of WPC on enzymatic hydrolysis and fermentation was greatly dependent on the WPC concentration and the pretreatment employed. AL pretreatment released the most quantity of phenolics, followed by DA, AFEX and LHW pretreatment. At the same phenolics concentration, the inhibitory effect of phenolics on enzymatic hydrolysis and fermentation varied significantly. The inhibitory effect of WPC on enzymatic hydrolysis followed the order: AFEX > LHW > DA > AL, while the inhibitory effect of WPC on *Z. mobilis* 8b fermentation followed the order: AL > LHW > DA > AFEX. Based on the characteristics of WPC, the strong inhibitory effect of AFEX-WPC on enzymatic hydrolysis and its low inhibitory effect on fermentation was likely related with the presence of phenolic amides. The strong inhibitory effect AL-WPC on fermentation was likely due to the high content of monomeric phenolic aldehydes. As both phenolic concentration and structural characteristics affected their inhibitory effects, the inhibitory effect of phenolics derived from AFEX on actual enzymatic hydrolysis of lignocellulosic biomass would be lower due to their low content in the AFEX-pretreated biomass. Overall, the present study provides a new insight into the phenolics-caused inhibition on both hydrolysis and fermentation, which could guide the design of effective pretreatment with low toxicity and efficient detoxification strategies that aim to selectively remove or modify the degradation products prior to enzyme hydrolysis and microbial fermentation.

## Materials and methods

### Biomass and enzyme

Corn stover biomass used in this study was harvested and air-dried in 2017 at Lianyungang, Jiangsu province, China. The corn stover was knife milled using a laboratory mill with 4 mm particle size interior sieve. The raw corn stover consists of 37.2 ± 0.7% glucan, 19.2 ± 0.0% xylan, 19.5 ± 0.1% Klason lignin, and 3.9 ± 0.5% ash. The corn stover biomass was then stored in plastic containers at 4 °C until further use. Cellulase (60 mg protein/mL enzyme preparation) was generously provided by Qingdao Vland Biotech Co., Ltd.

### Biomass pretreatment

AL pretreatment was performed in an autoclave with 10% (w/w) solid loading, 2% (w/w) NaOH, at 121 °C for 20 min [40]. DA pretreatment was carried out in a 2-L high pressure reactor. The reactor containing 10% (w/w) dry weight and 1% (w/w) of sulfuric acid was heated to 160 °C and maintained for 10 min [40]. LHW pretreatment was performed at 200 °C for 5 min with 10% (w/w) solid loading. After AL, DA and LHW pretreatment, the slurry (liquid stream and solid loading) was allowed to cool down to room temperature and were then centrifuged at 10,000 rpm for 10 min to obtain pretreatment liquid stream. AFEX pretreatment in this work was performed in a 1-L reactor (Anhui Kemi Machinery Technology Co., LTD). AFEX pretreatment conditions were 120 °C for 30 min at 60% moisture with the ratio of anhydrous ammonia to biomass (dry weight) equal to 1:1. After the treatment, the pretreated corn stover biomass was placed in a fume hood to remove residual ammonia, followed by adding 300 mL deionized water. After mixing, the slurry was centrifuged at 10,000 rpm for 10 min to separate pretreatment liquid stream. In all analyses above, pretreatment liquid stream (PLS) was neutralized to pH 7 and then subjected to compositional analysis.

### Extraction of water-soluble phenolic compounds (WPC) from PLS

To study the effect of WPC, ethyl acetate extraction was performed to extract phenolics from pretreatment biomass. This method has been commonly used in previous studies to extract phenolics from various natural resources [31, 41]. The pH of PLS was adjusted to pH 7 by 0.1 M of NaOH or HCl to protonate the phenolic acids and other aromatic components of the mixture so as to increase their solubility in ethyl acetate. Then, one volume of PLS was mixed with three volumes of ethyl acetate, and allowed to sit for 30 min for phase separation. Subsequently, the top ethyl acetate phase was collected and three volumes of fresh ethyl acetate were added into the bottom water phase for another round of extraction. The extraction was repeated three times and the collected ethyl acetate phase was concentrated through a rotary evaporator. After evaporation, the concentrated phenolics fraction was placed in fume hood for 48 h to remove residual solvent and it was then further freeze-dried to remove residual water. The freeze-dried phenolics was dissolved in deionized water (200 mL) at 50 °C, and centrifuged at 10,000 rpm for 10 min. The WPC was dried at 105 °C for 5 h, which was further used for mass balance analysis. The WPC was concentrated through a freeze drier, which was further used for composition analysis, mass balances, enzymatic hydrolysis and fermentation.

### Synthesis of *p*-coumaric amide

*p*-Coumaric amide was synthesized according to a reported method [42]. Specifically, 20 g *p*-coumaric acid and 150 mL ethanol was added to 250 mL round-bottom flask equipped with a magnetic stir bar. 10 mL acetyl chloride was added and stirred at room temperature for 12 h. Ethanol was removed in a rotary evaporator at 50 °C to obtain the substrate. 10 mL of the substrate was transferred to heavy-wall long flask containing 40 mL concentrated ammonium hydroxide with a magnetic stir bar and sealed with a polytetrafluoroethylene cap. The sealed flask was incubated at 95 °C in a heating oil bath covered for 12 h. The flask was cooled and then left open for ~ 8 h in a hood to allow evaporation of ammonium hydroxide. Under vacuum on a glass filter, the precipitated products (Containing *p*-coumaric amide) were collected. The purity of the products was analyzed by silica gel TLC developed with 4% methanol in chloroform. Only preparations exceeding 95% purity were used for further experiments.

### Enzymatic hydrolysis

To evaluate the effects of inhibitors from each pretreatment on enzymatic hydrolysis, Avicel (Purchased from sigma company) was enzymatically hydrolyzed using commercial cellulase or xylanase mixture at 2% w/w dry weight loading (pH 4.8, 50 °C) using a vertical rotator (25 rpm). The hydrolysis was carried out at 20 mg protein/g glucan loading with addition of 0 g/L, 2 g/L, 4 g/L, 6 g/L WPC derived from each pretreatment, and the pH was maintained at 4.8 with 50 mM citrate buffer. After hydrolysis, monomeric glucose and xylose concentrations of the liquid samples were determined by HPLC as described previously [40, 43].

### Fermentation

Medium containing 60 g/L glucose, 30 g/L xylose, 5 g/L yeast extract, 10 g/L tryptone and 0 g/L, 2 g/L, 4 g/L, 6 g/L WPC from each pretreatment was used as substrate to evaluate the effects of inhibitors on fermentation by a recombinant *Z. mobilis* 8b strain, which can consume both glucose and xylose. Fermentation was carried out in a 10-mL flask with a total reaction volume of 8 mL and initiated by inoculating *Z. mobilis* 8b strain to reach OD_600_ of 1.0. The concentrations of glucose, xylose and ethanol were determined by HPLC [27].

### Analysis methods

Biomass composition was analyzed according to NREL methods [44]. The concentrations of glucose, acetic acid, ethanol, HMF and furfural were determined using a Shimadzu HPLC system equipped with an Aminex HPX-87H column maintained at 60 °C and Shimadzu refractive index detector (RID). 5 mM sulfuric acid was used as an eluent at 0.6 mL/min and the injection volume was 15 μL [40, 45].

The total phenol concentrations were analyzed through Folin–Ciocalteu assay. In detail, 100 μL sample was added into a glass tube and then mixed with 3 mL of water and 250 μL Folin–Ciocalteu reagent. After 5 min, 750 μL Na_2_CO_3_ solution (20%, w/w) and 900 μL water were then added to bring the volume to 5 mL. The tube was incubated at 22 °C for 2 h. The concentration of total phenol was then determined by measuring the absorbance at 760 nm [22, 46].

LC–MS analysis of WPC was conducted using a Sciex 4600 Triple TOF mass spectrometer, Shimadzu column oven and CTC PAL auto-sampler, and was operated using electrospray ionization. Ultimate XB-C18 column (2.1 × 100 mm, 3 μm particles) was used together with deionized water containing 0.1% of formic acid (mobile phase A), CAN (mobile phase B) and column temperature held at 40 °C. The gradient for analysis of the extract was as follows: 5% B until 2 min, change from 5% to 70% B in 13 min, from 70% to 90% B in 5 min, from 90% to 5% B in 5 min and constant 5% B Until the end. The flow rate was constant at 0.4 mL/min and the injected volume was 10 µL [31].

Fourier transform infrared (FT-IR) spectra of WPC were obtained by a reported procedure [22]. Twenty scans for each sample were taken with a resolution of 2 cm^−1^ ranging from 400 to 4000 cm^−1^. The sample was prepared using the method of agate mortar crush before scanning.

## Supplementary information

**Additional file 1.** Additional figures and tables.

## Data Availability

All data generated or analyzed during this study are included.

## Abbreviations

WPC: Water-soluble phenolic compounds
DA: Dilute acid
AL: Alkaline pretreatment
AFEX: Ammonia fiber expansion
LHW: Liquid hot water pretreatment
PLS: Pretreatment liquid stream
BSTFA: *N*,*O*-Bis(trimethylsilyl)-trifluoroacetamide containing 1% trimethylchlorosilane
GC–MS: Gas chromatography–mass spectrometer
FT-IR: Fourier transform infrared
HPLC: High performance liquid chromatography
*Z. mobilis* 8b strain: *Zymomonas mobilis* 8b strain
