# Supplementary material for: Understanding the structural characteristics of water-soluble phenolic compounds from four pretreatments of corn stover and their inhibitory effects on enzymatic hydrolysis and fermentation
Source: Biotechnol Biofuels. 2020 Mar 11;13:44. doi: 10.1186/s13068-020-01686-z (PMC7065323; doi:10.1186/s13068-020-01686-z)
Supplement: Supplementary file 1 — Additional file 1. Additional figures and tables. [file 13068_2020_1686_MOESM1_ESM.docx]

Additional Information for:

**Understanding the structural characteristics of water-soluble phenolic compounds from four pretreatments of corn stover and their inhibitory effects on enzymatic hydrolysis and fermentation**

**Xiangxue Chen ^1,#^, Rui Zhai ^1,#^, Ying Li ^1^, Xinchuan Yuan ^1^, Zhi-Hua Liu ^2^, Mingjie Jin^1,*^**

^1^School of Environmental and Biological Engineering, Nanjing University of Science and Technology, 200 Xiaolingwei Street, Nanjing 210094, China.

^2^Department of Plant Pathology and Microbiology, College of Agriculture and Life Sciences, Texas A&M University, College Station, TX 77843, USA

***Corresponding author:**

Mingjie Jin, Tel/fax: +86-25-84315173; E-mail: jinmingjie@njust.edu.cn.

# These authors contributed equally to this work.

**Authors’ contributions**

Xiangxue Chen, Rui Zhai, Ying Li and Xinchuan Yuan performed the experiments, and analyzed the data; Mingjie Jin coordinated and supervised this study; Xiangxue Chen, Rui Zhai, Zhi-Hua Liu and Mingjie Jin drafted and revised the manuscript. All authors read and approved the final version.

**Supplementary Figures**





Figure S1. Effect of water-soluble phenolic compounds (WPC) on enzymatic hydrolysis of washed alkali pretreated corn stover. The washed alkali pretreated corn stover contained 54.7±0.12% cellulose, 21.8±0.6% xylan and 10.9 ± 0.7 % lignin. The hydrolysis was carried out at 20% w/w solid loading with an enzyme loading of 30 mg protein/g glucan (cellulase: xylanse=2:1) and with addition of WPC derived from each pretreatment. Citrate buffer (50 mM; pH 4.8 ) was used for enzymatic hydrolysis.

**Additional Tables**

Table S1.Chemical analysis of WPC (containing 2g/L phenolics) from AL, DA, LHW and AFEX pretreatment.

| Components in WPC extract (g/L) | Pretreatment methodology | | | | |
| --- | --- | --- | --- | --- | --- |
|  | AL^a^ | DA^a^ | LHW^a^ | AFEX^a^ | |
| Olig-glu^b^ | 0.01 | 0.10 | 0.03 | | 0.00 |
| Olig-xyl^b^ | 0.32 | 0.10 | 0.07 | | 0.21 |
| Glucose | 0.00 | 0.11 | 0.00 | | 0.01 |
| Xylose | 0.00 | 0.21 | 0.02 | | 0.00 |
| Acetic acid | 0.12 | 0.13 | 0.02 | | 0.04 |
| Furfural^c^ | 0.00 | 0.00 | 0.00 | | 0.00 |
| HMF | 0.00 | 0.01 | 0.00 | | 0.00 |

^a^:AL:alkali; DA:dilute acid; LHW: Lqiud hot water; AFEX: Ammonia fiber expansion.

^b^:Olig-glu: Oligoglucose; Olig-xyl:Oligoxylose.

^c^:Furfural concentrations were too low to be detected.

Table S2. LC-MS analysis of WPC derived from AL, DA, LHW and AFEX pretreatment.

|  | Compounds | Chemical formula | Percentage (%) |
| --- | --- | --- | --- |
| AL-CS | p-Coumaric acid | C9H8O3 | 54.55% |
|  | Benzaldehyde | C8H8O | 23.81% |
|  | Benzoic acid | C6H5COOH | 8.42% |
|  | Succinic acid | C4H6O4 | 4.51% |
|  | Syringic acid | C9H10O5 | 3.24% |
|  | Ferulamide | C10H11NO3 | 0.66% |
|  | Vanillin | C8H8O3 | 0.47% |
|  | Phenylalanine | C9H11NO2 | 0.18% |
|  | Decahexose | C10H10O6 | 0.15% |
|  | Catechol | C30H48O5 | 0.11% |
|  | Hydroquinone | C6H6O2 | 0.06% |
| AFEX-CS | p-Coumaric acid | C9H8O3 | 53.73% |
|  | Benzaldehyde | C8H8O | 21.57% |
|  | Ferulamide | C10H11NO3 | 18.62% |
|  | Syringic acid | C9H10O5 | 0.50% |
|  | Benzoic acid | C6H5COOH | 0.36% |
|  | Vanillin | C8H8O3 | 0.18% |
|  | Vanilla amide | C8H9NO3 | 0.13% |
|  | Catechol | C30H48O5 | 0.06% |
|  | Succinic acid | C4H6O4 | 0.05% |
| LHW-CS | Benzoic acid | C6H5COOH | 59.89% |
|  | Syringic acid | C9H10O5 | 6.65% |
|  | Vanillin | C8H8O3 | 2.85% |
|  | p-Coumaric acid | C9H8O3 | 1.59% |
|  | Succinic acid | C4H6O4 | 1.56% |
|  | Phenylalanine | C9H11NO2 | 1.06% |
|  | Decahexose | C10H10O6 | 1.05% |
| DA-CS | p-Coumaric acid | C9H8O3 | 61.87% |
|  | Benzaldehyde | C8H8O | 28.03% |
|  | Syringic acid | C9H10O5 | 5.11% |
|  | Benzoic acid | C6H5COOH | 0.76% |
|  | Vanillin | C8H8O3 | 0.72% |
|  | Hydroquinone | C6H6O2 | 0.40% |
|  | Decahexose | C10H10O6 | 0.31% |
|  | Succinic acid | C4H6O4 | 0.24% |
|  | Cinnamamide | C9H9NO | 0.06% |
|  | Catechol | C30H48O5 | 0.0% |

The percentage of identified phenolics was calculated based on the detected compounds; the unidentified compounds were not reported in this table.
